# Matrix assisted laser desorption ionization mass spectrometry imaging identifies markers of ageing and osteoarthritic cartilage

**DOI:** 10.1186/ar4560

**Published:** 2014-05-09

**Authors:** Mandy J Peffers, Berta Cillero-Pastor, Gert B Eijkel, Peter D Clegg, Ron MA Heeren

**Affiliations:** 1 Institute of Ageing and Chronic Disease, University of Liverpool, Leahurst, Chester High Road, Neston, Wirral CH64 7TE, UK; 2 Biomolecular Imaging Mass Spectrometry (BIMS), FOM Institute AMOLF, Science Park 104, 1098 XG Amsterdam, The Netherlands

## Abstract

**Introduction:**

Cartilage protein distribution and the changes that occur in cartilage ageing and disease are essential in understanding the process of cartilage ageing and age related diseases such as osteoarthritis. The aim of this study was to investigate the peptide profiles in ageing and osteoarthritic (OA) cartilage sections using matrix assisted laser desorption ionization mass spectrometry imaging (MALDI-MSI).

**Methods:**

The distribution of proteins in young, old and OA equine cartilage was compared following tryptic digestion of cartilage slices and MALDI-MSI undertaken with a MALDI SYNAPT™ HDMS system. Protein identification was undertaken using database searches following multivariate analysis. Peptide intensity differences between young, ageing and OA cartilage were imaged with Biomap software. Analysis of aggrecanase specific cleavage patterns of a crude cartilage proteoglycan extract were used to validate some of the differences in peptide intensity identified. Immunohistochemistry studies validated the differences in protein abundance.

**Results:**

Young, old and OA equine cartilage was discriminated based on their peptide signature using discriminant analysis. Proteins including aggrecan core protein, fibromodulin, and cartilage oligomeric matrix protein were identified and localised. Fibronectin peptides displayed a stronger intensity in OA cartilage. Age-specific protein markers for collectin-43 and cartilage oligomeric matrix protein were identified. In addition potential fibromodulin and biglycan peptides targeted for degradation in OA were detected.

**Conclusions:**

MALDI-MSI provided a novel platform to study cartilage ageing and disease enabling age and disease specific peptides in cartilage to be elucidated and spatially resolved.

## Introduction

Osteoarthritis (OA) is an age related joint disease characterized by alterations in the chondrocytes and loss of cartilage extracellular matrix (ECM) [1]. Age is the most common risk factor for its initiation and progression, with symptomatic OA affecting 10 to 20% of people more than 50 years old [2]. ‘Wear and tear’ injuries due to mechanical loading over many years, genetic polymorphisms and other factors contribute to the development of the disease.

Although much work has been undertaken to investigate the pathogenesis of OA the molecular mechanisms involved are not fully understood, with few validated markers for disease diagnosis and progression being available. Treatment of OA is limited to pain relief with few disease-modifying therapeutics available to patients. Hence, methodologies which permit the detailed study of aging and OA cartilage organization could improve the knowledge of how aging increases the risk of OA. Previously the comparison of young, old and OA tissue has been challenging as the provision of normal old and old OA is difficult and few markers have been identified that differentiate between age or disease-related change in cartilage except urinary collagen type II [3].

Measurement tools enabling the capture of the dynamic and complex interplay between proteins, lipids, DNA and other molecules are necessary. This interplay involves the appearance, interaction and disappearance of many species on varying time scales. Capturing this spatial and temporal information will help in the quest to understand the pathogenesis of OA and ultimately provide disease modifying treatments. Mass spectrometry (MS) is an analytical tool that enables the accurate measurement of the mass-to-charge ratio of ions. Matrix assisted laser desorption/ionization mass spectrometry imaging (MALDI-MSI) of tissue samples is a powerful technique that allows for spatially resolved, comprehensive and specific characterization of hundreds of unknown molecular species (proteins, peptides, lipids or metabolites) *in situ* in a single molecular imaging experiment [4]. The label-free nature of the technique combined with high spatial resolving power better than 50 micrometer makes it an ideal tool to study the molecular aspects of aging in cartilage tissue [5]. Specific protein patterns revealed by MSI have been demonstrated as prognostic [6] and diagnostic indicators [7]. MSI techniques have only recently been implemented in cartilage research. One study employed a time of flight secondary ion mass spectrometry (TOF-SIMS) workflow in order to acquire molecular-specific spatial distribution of lipids in normal and OA cartilage [8]. A further study used MALDI-MSI to identify and study OA specific peptides and proteins in cartilage [9]. In this and other works, a direct correlation between the detected peptides and the distribution and identity of the original precursor protein was achieved [10].

The horse provides an excellent model for the study of cartilage in man as it suffers clinical joint diseases similar to man [11] and, as such, has been used as a model of naturally occurring OA [12] due to the extensive knowledge of its pathogenesis and clinical experience of the disease [13]. Indeed, the incidence of equine metacarpophalangeal OA in young racehorses [14] in training is similar to the incidence of post traumatic OA in man [15]. Additionally, the articular cartilage thickness is also comparable between the species [16].

The aim of this study was to establish peptide profiles in young, aging and OA horse cartilage of full thickness cartilage from surface to bone, with a high spatial distribution to determine changing molecular events distinct between aging and disease. We hypothesize that many alterations in specific peptide abundance are due to proteinase driven degradation.

## Methods

### Sample collection, preparation and processing

Ethical approval was not required for this study as samples were obtained from an abattoir. Full thickness equine cartilage slices were removed from the mid condyle region of metacarpophalangeal joints collected from an abattoir and snap frozen in liquid nitrogen within hours. All samples were scored macroscopically using Kawcak scoring for pathological grading of the distal condyles of metacarpophalageal III [17]. For age-related studies, samples were taken from skeletally mature young (four years old, n = 3) and old (greater than fifteen years old, n = 3) horses. For OA studies, old normal samples were taken from macroscopically and microscopically normal condylar areas adjacent to diseased tissue (n = 3) (greater than 15 years old). For young horses, one year is equivalent to an age of approximately 3.5 years of a human [18]. Hence, horses more than 15 years old used in this study equate to humans of older than 52 years. For OA studies, skeletally mature donors >15 years were chosen with mild macroscopic OA changes.

Cartilage was sectioned perpendicular to the joint surface at 12 μm thicknesses on a cryostat Microm HM 525 (Microm International, Walldorf, Germany) and thaw mounted on glass slides. All samples were dried in a vacuum desiccator for 10 minutes prior to further processing.

### Tissue digestion, matrix deposition and MALDI-MSI

Young and old cartilage was studied in duplicate while old and OA cartilage was examined in triplicate and then compared by MALDI-MSI. The sections were washed in 70% ethanol and chloroform for 30 seconds each. Tissue digestion and matrix deposition and MALDI-MSI were undertaken as previously described [9].

### Multivariate analysis and data interpretation following MALDI-MSI

Data pre-treatment, principal component analysis (PCA) and discriminant analysis (DA) were performed using AMOLF in-house build MATLAB (The MathWorks, Natick, MA, USA) software tools [9]. The first 20 principal components (PC) were used as input for discriminant analysis (DA). PCA was performed on the raw spectral data and was used as a data compression and noise filtering step before application of DA. In short, PCA is an unsupervised statistical method that aims at pooling together a maximum amount of variance in a minimum number of independent variables. These new variables (principal components) are a construct of the original variables (mass channels) weighed by their correspondence with the new variable. This weighing factor is referred to as the PC loading. DA is a supervised multivariate method that calculates the combination of variables (in this case PCs) which gives maximum separation between the pre-defined groups. DA maximizes the between group - within group variance.

Biomap 3.7.5.5 software (Novartis Pharma AG, Basel, Switzerland) was used to generate ion images. Normalization of the intensity of all *m/z* channels was performed using the intensity of the m/z 190 matrix peak. *P* values for statistical differences found in MALDI-MSI experiments were calculated with one-way analysis of variance (ANOVA) with a Bonferroni *post hoc* test using SPSS (SPSS Inc. Chicago IL, USA) following normality testing. Differences were considered to be statistically significant at *P* ≤0.05. The data were expressed as mean intensity ± standard error.

### Tissue digestion and matrix deposition for profiling experiments

Protein identification was undertaken with profiling experiments directly from each donor tissue by applying 10 μL of trypsin diluted in 25 mM ammonium bicarbonate 0.05 μg/μL directly and incubating overnight at 37°C. α-Cyano-4-hydroxycinnamic acid (CHCA) matrix was applied as previously described [9]. Data dependent analysis (DDA) of tryptic peptides was performed with the MALDI SYNAPT™ HDMS system. Every MS survey scan was followed by collisional fragmentation of the most intense ions with subsequent collection of tandem mass spectroscopy (MS/MS) spectra. Direct MS/MS fragmentations were performed directly from the tissue on the peptides that differentiate young, old or OA tissue. These target peptides were found following DA. In addition, fragmentation was also undertaken on a peptide list generated from bibliographical analysis of previous MALDI-MSI studies in cartilage [9]. The resulting data files were submitted to an in-house Mascot server (Matrix Sciences, London, UK) and searched against the Unihorse and Swissprot databases. Search parameters used were: peptide mass tolerances 50 ppm, fragment mass tolerance of 0.5 Da, 1+ ions, missed cleavages; 3, and instrument type MALDI-Q-TOF. Modifications used were variable oxidation of methionine.

### Histological staining and immunohistochemistry studies

Following MSI the tissue sections were washed in 70% ethanol for five minutes to remove matrix. The slides were immersed in Harris hematoxylin solution (Sigma-Aldrich, Dorset, UK) for one minute. After washing with water for 15 minutes, they were rinsed in 95% ethanol and counterstained in an eosin solution for 30 seconds. Digital images were acquired with the Mirax system (Carl Zeiss, Sliedrecht, The Netherlands) after dehydrating steps. A score based on the modified Mankin’s scoring system for semi-quantitative histological assessment of all equine cartilage samples was undertaken [19].

Macroscopic grading was used to allocate samples into normal or OA groups, and scores of greater than 0 were assigned OA. Only macroscopically normal cartilage was used for age related studies. All samples for age related studies had a modified Mankin’s grade of 0 (data not shown). The macroscopic grading and Mankin’s score for each sample used in the old normal versus OA studies are shown in Additional file 1: Table S1.

To perform immunohistochemistry studies, paraffin-embedded tissues were cut into 4 μm sections by a microtome. Protease antigen retrieval was undertaken with a bacterial protease solution. Sections were incubated with polyclonal rabbit anti-human fibronectin (Dako, Heverlee, Belgium) at 1:2400 dilution, overnight at 4°C. Goat anti-rabbit biotinylated secondary antibody at 1:100 was incubated at room temperature for 30 minutes. Immunostaining was detected with avidine-biotin complex (Vector Laboratories, Peterborough, UK) and chromogenic substrate diaminobenzidine (Sigma-Aldrich, Dorest, UK). Counterstaining was undertaken with Papanicolau stain (Merck Millipore, Watford, UK). Negative control experiments were carried out with omission of the primary antibody and substitution with non-immune rabbit immunoglobulin G (IgG) (Abcam, Cambridge, UK). No staining was observed in negative control experiments. Digital images were visualized and acquired with a Nikon DS-L2 stand-alone control unit using a Nikon eclipse 80i microscope. Analysis was undertaken with ImageJ. ImageJ 1.42 image software and band densitometry was analyzed by arbitrarily drawing a rectangular box around the selected protein bands of interest. The same rectangular box was used to measure all bands of interest and band intensities were quantified by conversion to profile plot histograms. By specifically selecting the peak of interest and closing off the peak with the line tool, background-subtracted density of each peak of interest is quantified. All densitometry data are normalized to the band intensity of the loading control used.

### Proteoglycan-enriched fraction isolation from cartilage

In order to validate our hypothesis that the reduction in specific peptides identified by MALDI-MSI was due to degradation of those specific peptides we undertook proteinase digestion of proteoglycan-enriched cartilage. Equine articular cartilage from the grossly normal metacarpophalangeal joint of three horses, mean age 10 ± 1 years, obtained from an abattoir was pooled and pulverized with a dismembranator (Miko, S-Braun, Bethlehem, PA, USA). Proteins were extracted with cartilage extraction buffer containing 4 M guanidinium chloride, 50 mM sodium acetate and proteinase inhibitors (complete protease inhibitors, ethylenediaminetetraacetic acid (EDTA)-free, Roche, Lewes, UK), pH 6.0 using end-over-end mixing for 20 hours at 4°C. After extraction the soluble fraction (proteoglycan-enriched) was removed following centrifugation for 15 minutes at 13,000 g at 4°C. Following dialysis in a 14,000-kD cut-off membrane (Spectrapor, Breda, NL) for 24 hours at 4°C against 0.1 M sodium acetate, pH 6.0 in the presence of proteinase inhibitors, the extract was clarified using centrifugation for 15 minutes at 13,000 g at 4°C. A crude proteoglycan-enriched extract was isolated by associative cesium chloride (CsCl) density gradient centrifugation [20], also in the presence of the proteinase inhibitors. The supernatant was fractionated in an associative CsCl density gradient (starting density 1.5 g/ml) for 60 hours at 100,000 g in an ultracentrifuge (Beckman 50Ti, Galway, Ireland). The tube was fractionated into quarters, A1-A4 [21], and the A1-A2 fraction dialyzed first against 0.1 M sodium acetate for 48 hours at 4°C and then against ultrapure water for 36 hours at 4°C, both in the presence of proteinase inhibitors. The samples were then lyophilized.

### Proteinase digestion of the proteoglycan-enriched fraction *in vitro*

A reconstituted A1-A2 aliquot was digested in proteinase digestion buffer (50 mM Tris HCl, 100 mM NaCl, 10 mM CaCl2, pH 7.5) with 0.014 nmol truncated human recombinant ADAMTS-4 (a distintegrin and metalloproteinase with thrombospondin motifs 4) (Calbiochem, La Jolla, CA, USA) for seven hours at 37°C. A control aliquot was incubated under the same conditions in the presence of recombinant protein formulation buffer. The enzymatic digestion reactions (1 ml) were stopped by addition of EDTA.

### In-solution tryptic digestion

The crude proteoglycan extracts following from the proteinase digestion were easily trypsin digested as previously described [22]. Samples were desalted and purified using C_18_ resin in the form of a ZipTip® (Merck Millipore, Rockland, MA, USA).

### LC-MS/MS analysis of proteinase digested cartilage proteoglycan-enriched fraction

Liquid chromatography (LC)-MS/MS analysis was performed using NanoAcquity™ Ultraperformance LC (Waters, Manchester, UK) on line to an LTQ-Orbitrap Velos (Thermo-Fisher Scientific, Hemel Hempstead, UK) as described previously [22].

### Data analysis proteinase digested cartilage proteoglycan-enriched fraction

In order to identify potential cleavage sites of ADAMTS-4 identification raw spectra were converted to mascot generated files (mgf) using Proteome Discoverer software (Thermo, Hemel Hempstead, UK). The resulting mgf files were searched against the Unihorse database using an in-house Mascot [23] server (Matrix Science, London, UK). Search parameters used were: enzyme; none, peptide mass tolerances 10 ppm, fragment mass tolerance of 0.6 Da, 1+, 2+ and 3+ ions, missed cleavages; 1, and instrument type ESI-TRAP. Modifications included were: fixed; carbamidomethyl cysteine and variable; oxidation of methionine.

Patterns of fragmentation were determined for biglycan and fibromodulin. The probability that a match was correct (*P* <0.05) was determined using the Mascot derived ion score where *P* was the probability that the observed match was a random event.

To validate the results further, raw data files were loaded into PEAKS® Studio 6.0 (Bioinformatics Solutions Inc., Waterloo, Canada) and *de novo* sequencing and protein identification performed. PEAKS® software employs multiple analytical algorithms and is able to identify every peptide in the data, and can validate database searches using *de novo* sequencing results. Estimate false discovery rate (FDR) function was used in order to create a ‘decoy fusion’ database based on the Unihorse database. Data prerefinement was undertaken by choosing peak centroiding, charge deconvolution, and deisotope options. The quality value was set greater than 0.65. The search parameters for the PEAKS® software were identical to those for the Mascot search. Results generated using PEAKS® Studio were manually curated against the MASCOT search engine results. Only peptides with a 10lgP score of >20 were considered as significant [24].

## Results

### Peptide identification

MS/MS profiling experiments on cartilage slices were undertaken in order to determine proteins that could be identified directly from the tissue (Table 1). Tryptic peptides of cartilage oligomeric matrix protein (COMP) and fibromodulin were identified in all groups. Following PCA and DA between young, old and OA groups, young and old, and old and OA, the peaks with the highest absolute loadings in the DF1 spectra were targeted directly from tissue slices for MS fragmentation and database searching in order to identify the protein it pertained to. In addition, a list of masses was also selected that had been previously identified in human cartilage MALDI-MSI studies (Table 1) [9].

**Table 1:** Proteins identified from MS/MS profiling and targeted MS experiments of cartilage

**Accession**	**Protein**	**Observed mass**	**Score from profiling MS**	**Score from targeted MSMS**	**Peptide**	**Identified targeted MS studies**
O18832_HORSE	Aggrecan	2,325.3		43	R.VSLPNYPAIPTDATLELQNLR.S	✓
PGS1_HORSE	Biglycan	1,312.8	41	43	K.IQAIELEDLLR.Y	✓
PGS1_HORSE	Biglycan	2,027.2		47	K.NHLVEIPPNLPSSLVELR.V	✓
CILP2_HORSE	Cartilage intermediate layer protein	2,196.2	96	67	R.FLPSEQIQGVVVSAINLEPR.A	✓
COMP_HORSE	Cartilage oligomeric matrix protein	1,698.9	31		K.QMEQTYWQANPFR.A	
COMP_HORSE	Cartilage oligomeric matrix protein	2,256.1		86	R.FYEGPELVADSNVVLDTTMR.G^a^	✓
CHAD_BOVIN	Chondroadherin	2,198.3	17		R.AGAFDDLTELTYLYLDHNK.V	✓
CO2A1_MOUSE	Collagen alpha-1(II) chain	1,679.9	39	75	K.DGETGAAGPPGPSGPAGER.G	✓
CO2A1_HORSE	Collagen alpha-1(II) chain	1,427.9	23		K.ALLIQGSNDVEIR.A	
CL-43_BOVINE	Collectin 43	2,415.0		33	K.GEPGPEGGVGAPGMPGSPGPAGLKGER.G	✓
FMOD_BOVIN	Fibromodulin	1,557.0	67	42	R.SLILLDLSYNHLR.K	✓
FMOD_BOVIN	Fibromodulin	1,955.2	67	74	K.IPPVNTNLENLYLQGNR.I^a^	✓
FINC_HORSE	Fibronectin	1,401.7		68	K.HYQINQQWER.T^a^	✓
HBBA_HORSE	Hemoglobin subunit beta-A	1,274.9	23		R.LLVVYPWTQR.F	
MATN3_HORSE	Matrilin-3	1,618.0	20		K.SRPLDLVFIIDSSR.S	
MIA3_MOUSE	Melanoma inhibitory activity 3	1,186.7	24		R.EYAPGVLPGKR.D	

The proteins identified following profiling and targeted MS studies from cartilage are represented. The table identifies the Swissprot accession number, abbreviation of the protein name, experimental molecular weight of the matched peptides, the score given by the Mascot algorithm and the sequence of the matched peptides. All Mascot scores were significant (*P* <0.05). All peptide sequences were checked against the equine sequence and were homologous. ^a^denotes peptides previously identified in a human cartilage MALDI-IMS study [9]. MS, mass spectrometry; MS/MS, tandem mass spectroscopy.

### MALDI-MSI reveals the peptide signature in young, old and OA cartilage

#### Young, old and OA cartilage

After imaging experiments, data from young, old and OA cartilage groups were analyzed together. The spectra of each group revealed different profiles. A combined spectrum of representative digested equine young, old and OA samples is demonstrated in Additional file 2: Figure S1. PCA analysis and a scatter plot of DF1-DF2 revealed differences between each sample type (Additional file 3: Figure S2A and B). In addition, a comparison between biological repeats of the same subgroup was undertaken to assess the reproducibility of the score distribution of independent samples (data not shown).

**Figure 1:**
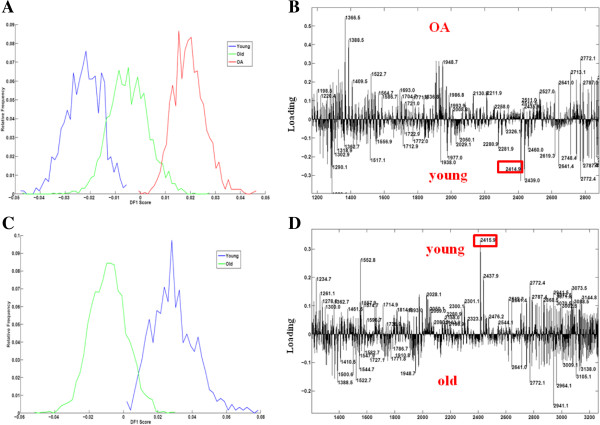
**PCA and DA of young, old and OA cartilage. A)** The spectra of all the old and OA samples after MALDI-MSI experiments were analyzed by PCA and DA to classify peptides specific of each category using a 1 D plot. **B)** Loading plot of DF1 representing the peptides specific to OA given by MALDI-MSI. The young specific peak 2415.9 is ringed. Figure C and D. PCA and DA separated the dataset on age of the sample. **C)** The spectra of all the young and old samples after MALDI-MSI experiments were analyzed by PCA and DA to classify peptides specific to each age using a 1 D plot. **D)** Loading plot of DF1 representing the peptides specific to age of cartilage given by MALDI-MSI. The young specific peak *m/z* 2414.9 is ringed. DA, discriminant analysis; MALDI, matrix assisted desorption ionization; MSI, mass spectrometry imaging; OA, osteoarthritis; PCA, principal component analysis.

Following DA a number of peaks were detected as specific for young, old or OA groups (Figure 1A). A peptide specific to young samples with *m/z* 2414.9 was subsequently identified as collectin-43 using fragmentation targeting of this mass (Figure 1B).

In addition, comparisons were also made using PCA and DA between young and old, and old and OA groups separately.

#### Young and old cartilage

Differential peptide distributions between young and old cartilage were assessed by MALDI-MSI followed by DA. The resulting DF1 classified the data into young and old (Figure 1C). The DF1 spectra (Figure 1D) identified that the peaks of the positive part of the DF1 were specific from young cartilage samples, such as collectin-43, and the peaks of the negative part were more abundant in cartilage from old donors.

**Figure 2:**
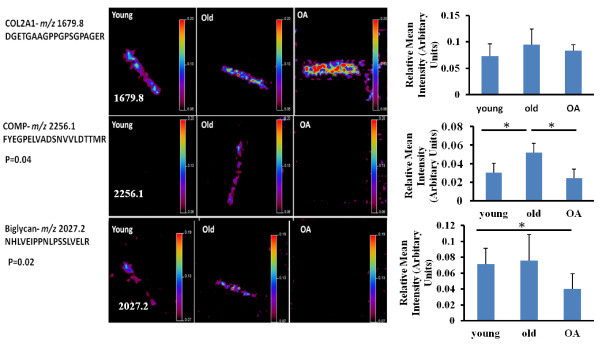
**Significant differential imaging of some ECM peptides.** Biomap was used to localize and quantify the differences in the peptide peak intensity of *m/z* 1679.8, *m/z* 2256.1 and *m/z* 2027.2 between young, old and OA cartilages. Samples representative of each group are illustrated here. Scale bar shows normalized intensities to 190 *m/z* matrix peak. In the scale bar red represents the highest signal. Histograms show the means of the relative peak intensities and 95% CI, n = 3 for each peptide. There was no change in the distribution of the type II collagen peak *m/z* 1679.8. However, a significant difference was evident for the COMP peptide *m/z* 2256.1 (*P* = 0.03 young versus old and old versus OA) and the biglycan peptide *m/z* 2027.2 (*P* = 0.02, young versus OA and old versus OA). COMP, cartilage oligomeric matrix protein; ECM, extracellular matrix; OA, osteoarthritis; 95% CI, 95% confidence interval.

The peak distribution intensity differences in young, old and OA cartilage of the identified peptides in Table 1 were analyzed by Biomap software. While there was no difference in the intensity of the majority of the peptides between groups, including collagen type II, a significantly different intensity of peptides for COMP, *m/z* 2256.1 and biglycan 2027.2 was observed (Figure 2).

#### Old and OA cartilage

**Figure 3:**
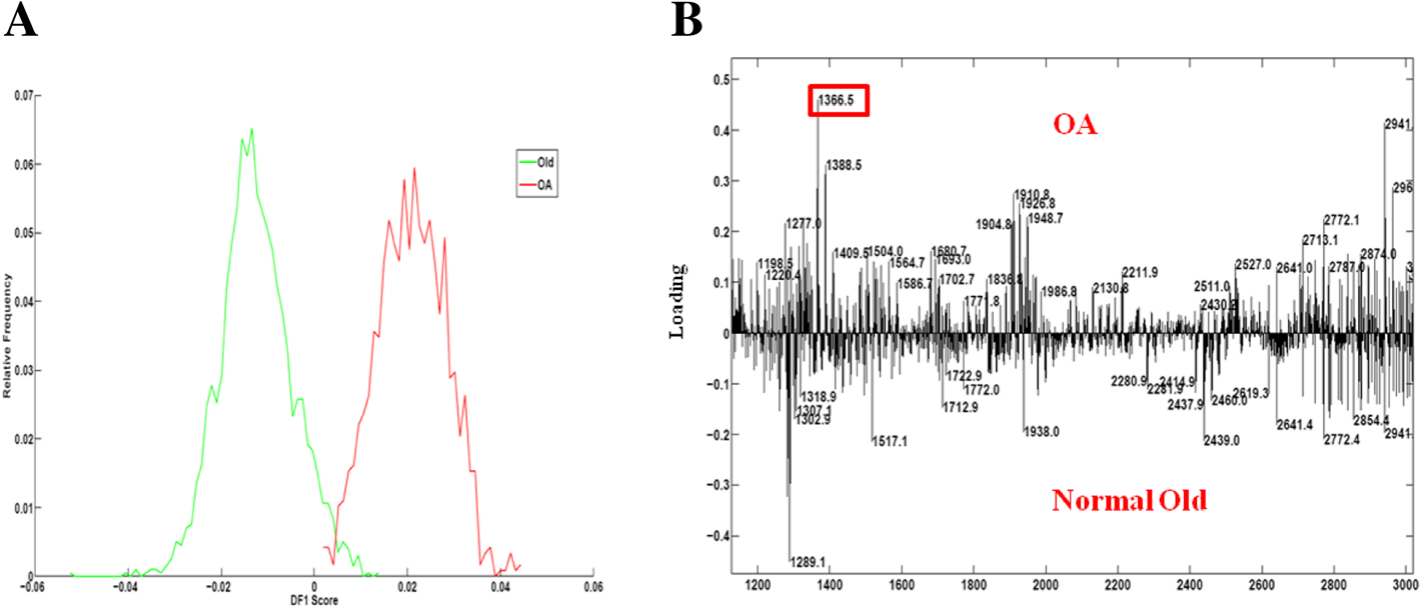
**PCA and DA of normal and OA cartilage. A)** The spectra of all the old and OA samples after MALDI-MSI experiments were analyzed by PCA and DA to classify peptides specific to each category using a 1 D plot. **B)** Loading plot of DF1 representing the peptides specific to OA given by MALDI-MSI. The OA specific peak 1366.5 is ringed. DA, discriminant analysis; MSI, MALDI, matrix assisted desorption ionization; OA, osteoarthritis; PCA, principal component analysis.

The data were assigned to two groups (OA and old) and DA was performed (Figure 3). The positive part of DF1 showed the masses specific to OA, the negative part the masses specific to the old group.

**Figure 4:**
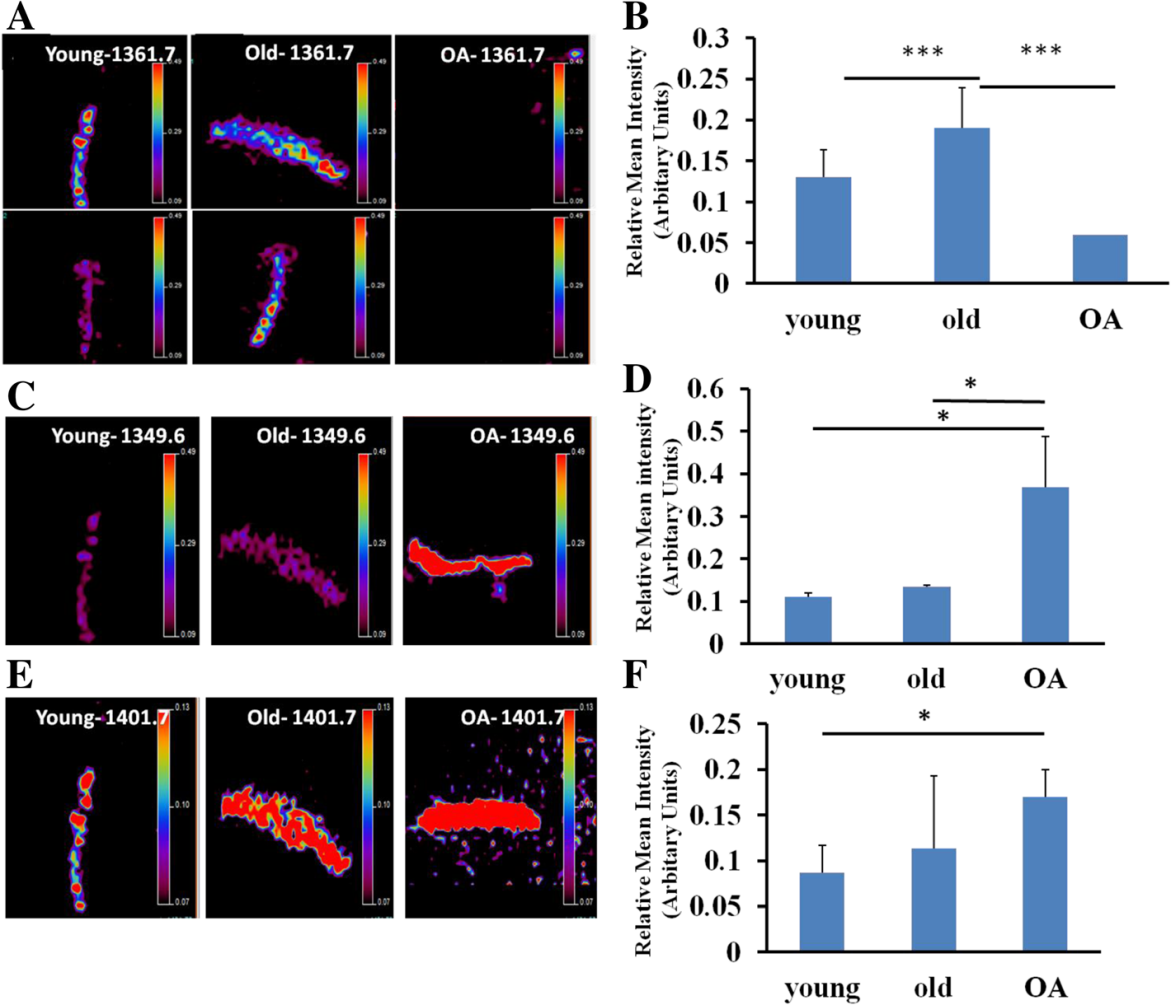
**Relative mean peak intensity of *m/z *1361.7, *m/z *1349.7 and *m/z *1401.7.** Biomap was used to quantify the differences in the peptide peak intensity of **A)***m/z* 1361.7, **C)***m/z* 1349.7 and **E)***m/z* 1401.7 between young, old and OA cartilages. For *m/z* 1361.7 two donors representative of each group are illustrated here. Scale bar shows normalized intensities to 190 *m/z* matrix peak. Histogram of **B)***m/z* 1361.7, **D)***m/z* 1349.7 and **F)***m/z* 1401.7 shows the means of relative peak intensities and 95% CI, n = 3, *represents *P* <0.05 and ***represents *P* <0.001. OA, osteoarthritis; 95% CI, 95% confidence interval.

**Figure 5:**
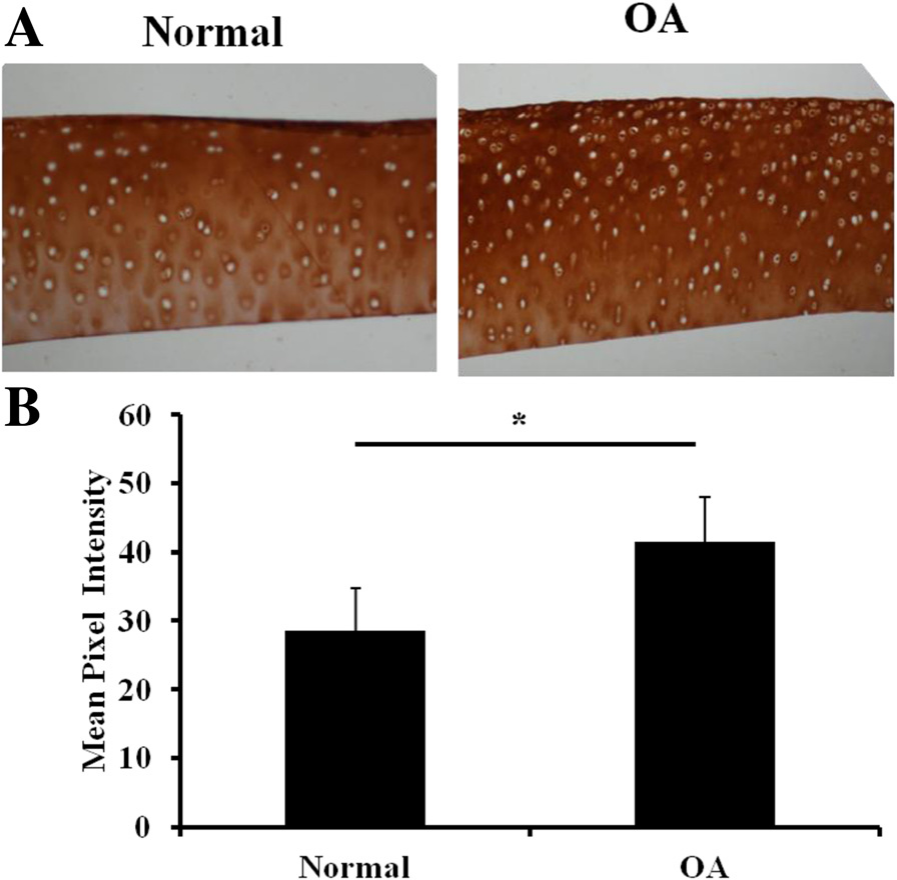
**Fibronectin validation in normal and OA cartilage. A)** A representative experiment of fibronectin immunohistochemistry in normal and OA samples is shown. **B)** Quantitation of differences in fibronectin staining between normal and OA cartilages (mean pixel intensity and 95% CI, n = 3; *P <0.05). OA, osteoarthritis; 95% CI, 95% confidence interval.

Of these, peptide *m/z* 1366.5 was found to be imaged with Biomap at greater intensities in all OA samples (mean intensity 1.01 ± 0.03), *P* <0.001, young or old versus OA). Mean intensity for young was 0.05 ± 0.003 and old 0.06 ± 0.04 (Additional file 4: Figure S3). In addition, we checked other peptides as found in other studies on human samples [9]. Distinctive intensity differences were observed for the previously identified fibromodulin peptide ELHLDHNQISR, *m/z* 1361.7. This peptide is homologous to the horse and was significantly reduced in OA equine cartilage (Figure 4A and B). Other peptides, such as *m/z* 1349.7, identified previously as a fibronectin peptide and homologous to the horse sequence were visualized. The highest intensity differences were between young (mean intensity 0.11 ± 0.009 and OA (mean intensity 0.36 ± 0.12) samples (*P* = 0.018, young versus OA). However, the intensity between old (0.12 ± 0.007) and OA was also significantly different (Figure 4C and D). Interestingly, a peptide *m/z* 1401.7 identified as fibronectin and previously nominated as a human OA marker [9] was visualized in young, old and OA samples (Figure 4E and F). The highest intensity differences for this peptide were between young (mean intensity 0.09 ± 0.009) and OA (mean intensity 0.17 ± 0.11) samples (*P* = 0.02, young versus OA). The higher intensity of fibronectin peaks in OA cartilage identified by MSI was orthogonally validated using fibronectin immunohistochemical staining (Figure 5). There was an increase in fibronectin staining in OA cartilage compared to old cartilage (mean pixel intensity 29.5 ± 1.9 old versus 41.5 ± 1.9 OA, n = 3, *P* <0.05). Primary and secondary antibody controls indicated that the primary antibody was specific to the antigen and the label was specific to the primary antibody (data not shown).

Finally, we measured the intensity of the distribution of peptides in different areas of the cartilage in young, old and OA cartilage in order to assess if the distributions were homogenous. However, we did not find differences in peptide distribution.

### Validation of fibromodulin and biglycan fragmentation patterns

In order to validate our MALDI-MSI data and test our hypothesis that alterations in specific peptide abundance identified by Biomap were due to proteinase driven degradation, we identified ADAMTS-4 derived fragmentation patterns in fibromodulin and biglycan. A number of ADAMTS-4 derived neopeptides were identified for fibromodulin and biglycan which may account for the reduction in peptide intensity identified by Biomap (Table 2).

**Table 2:** A number of ADAMTS-4 derived of biglycan and fibromodulin were identified with LC-MS/MS using MASCOT and PEAKS®

**Protein**	**Amino acid preceeding peptide residue**	**Peptide sequence identified**	**Amino acid residue after**	**PEAKS derived 10lgP**
Biglycan	**N**	**C**IEMGGNPLENSGFQPGAFDGLK	L	62.49
	K	NHLVEIPPNLP**S**^a^	**S**	42.42
	K	DLPETLNELHLDH**N**	**K**	59.46
Fibromodulin	R	DCPQECDCPPNFPTAM**Y**	**C**	53.23
	R	INEFSISSF**C**	**T**	29.37
	R	KVPDGLPSALEQLYLEHNNV**Y**	**S**	29.91
	R	ELHLDH**N**^a^	**Q**	28.85
	R	LSHNSLTN**N**	**G**	28.47
	R	KVPDGLPSALEQLYLEHNNVYSVPDS**Y**	**F**	29.91
	K	IPPVNTNLE**N**	**L**	20.78

^a^denotes a neopeptide indicating a probable cleavage site within a tryptic peptide identified in the MALDI-MSI experiment. Amino acids in bold indicate that the cleavage site is between these amino acids. ADAMTS, A distintegrin and metalloproteinase with thrombospondin motifs; LC, liquid chromatography; MS/MS, tandem mass spectrometry.

## Discussion

MALDI-MSI technology has been previously used in biomarker discovery [25] and to localize proteins, peptides and lipids in normal and OA human cartilage [8,9]. A recent study used MALDI-MSI to investigate synovial tissue from OA and rheumatoid arthritis patients resulting in the identification of differentially expressed proteins between the two diseases [26]. While these studies were undertaken on cartilage from normal and late OA adult human knees, nothing has been described until now in relation to age-related changes in cartilage and MALDI-MSI. In addition, this is the first time potential biomarkers of age and OA have been identified in cartilage using this technology.

Following MALDI-MSI and DA the peptide profile of young, old and OA horse cartilages could be distinguished. There was also a marked difference between the peptides identified in old versus OA as well as young versus old groups. Interestingly, when DA was applied to all three groups together (young, old, OA) there was a contribution of the old samples to young and OA indicating that peptides within old samples were also present in young and OA samples. This is not surprising as the majority of the proteins in young, old and OA cartilage will be the same ECM proteins [27]. Furthermore, previous studies have shown that tissue classified as OA still contains areas or cells with a healthy signature [8,9]. When a peak profile comparison was made between old and OA a distinction between OA related peptides was made which suggests that some age-related changes in peptides occur which do not contribute to disease related changes.

The major findings in this study were the identification of potential degradation sites in OA cartilage in biglycan and fibromodulin together with a number of potential markers of both age-related and disease-associated changes in cartilage. In addition, potential degradation sites within specific COMP peptides were identified. COMP is a major protein with a role in cartilage structural integrity through its collagen I, II, IX and chondrocyte binding capacity, interaction with other ECM proteins including matrilin-3 [28] and role in fibrillogenesis [29]. It has been proposed as a biomarker for arthritis [30]. Interestingly, the peptides identified were from the collagen binding C-terminal of COMP which others have demonstrated is important in intra- and extracellular processes [31]. Indeed, mutations in the genes encoding COMP and matrilin-3 result in multiple epiphyseal dysplasias [31,32]. Measurement of intact COMP and fragments thereof in synovial fluid or serum correlates to cartilage destruction in rheumatoid arthritis (RA) and OA patient studies [33]. Interestingly, studies in rats have found that the plasma levels of COMP are age dependent [34]. We observed a decrease in both young and OA compared to old cartilage in the distribution of the COMP peptide FYEGELVADSNVVLDTTMR. This peptide is located in the C-terminal end of COMP which binds collagen I, IX and II, and regulates fibril formation [29]. Our previous studies following cytokine stimulation of mature equine cartilage explants identified a neopeptide which indicated degradation of this peptide at Asn^712^–^713^Thr (data not shown). This suggests that in OA there is degradation within this tryptic peptide resulting in its reduced expression, demonstrated when OA cartilage was imaged. In young cartilage it may represent reduced synthesis or cartilage remodelling. The peptide represents a possible marker of age, but not disease, related changes in cartilage ECM. Future studies will investigate this finding further with the use of specifically designed monoclonal antibodies. It should be noted that MS intensities do not always reflect analyte concentration. It is possible that alterations in the nature of the tissue in aging and disease could influence ionization efficiency and, hence, cause ion suppression effects [35]. However, as our previous studies identified a degradation product within this peptide, this is less likely.

In addition, a marker of young cartilage, peak 2414.9 identified as collectin-43, was demonstrated. Collectin-43 is a C-type serum lectin with collagenous regions and a member of the collectin family of soluble proteins that are effector molecules in innate immunity [36]. Biglycan and decorin have been identified as binding collectin-43 and may have an important role in the resolution of C1q-mediated inflammatory processes in cartilage. Biglycan and decorin may down-regulate proinflammatory effects mediated by the collectins [37].

The study also demonstrated a reduction in some peptides for biglycan and fibromodulin, in both young and old compared to OA cartilage. Biglycan and fibromodulin are members of the small leucine rich repeat proteoglycan family with important collagen binding properties [38,39]. Structural changes related to aging are evident in biglycan, where there appears to be a cleavage in the amino terminal domain resulting in a ‘no-glycan’ biglycan as the terminal peptide containing the glycosaminoglycan chain separates from the protein core [40]. In order to study these findings further a cartilage-derived proteoglycan-enriched fraction was treated with the recombinant aggrecanase ADAMTS-4 and subjected to trypsin digestion and LC-MS/MS. In OA, the degradation of cartilage is characterized by a loss of ECM caused by secreted proteases, principally matrix metalloproteases and aggrecanases (reviewed [41]). We hypothesized that a reduction in the intensity of the fibromodulin tryptic peptide ELHLDHNQISR *m/z* 1361.7 and the biglycan mid-region tryptic peptide NHLVEIPPNLPSSLVELR *m/z* 2027.2 in OA samples was due to proteinase driven fragmentation within the peptide. The reduction in intensity of these peptides either represents a reduction in the synthesis, degradation from disease or differences in ionization. However, as another peptide identified in biglycan IQAIELEDLLR *m/z* 1312.7 (also mid-region) does not reveal reduced intensity in OA it is more likely that it is lost following degradation. Thus in OA, it is likely that there is cleavage within these tryptic peptides resulting in a loss of this mass and so a reduction in peak intensity.

Fibromodulin peptides were identified and their distribution imaged. The peptides m/z 1955.2 and 1361.7 correspond to the fibromodulin tryptic peptides identified in our human OA MALDI-MSI study [9]. Discrepancies in the abundance of the peptides may be due to the stage of OA in the two different studies. In the human study, the OA samples represented late stage OA while here we used lower grade early OA samples. One of a number of peptides, identified in MALDI-MSI studies of human cartilage [9], and with the same mass in the horse investigated was the fibromodulin peptide ELHLDHNQISR *m/z* 1361.7. This was significantly reduced in OA samples despite the distribution of other fibromodulin peptides identified being unchanged, for example with *m/z* 1557.0 or 1955.2 identified in profiling experiments between categories by either DA or following peak intensity analysis and statistical testing (data not shown). This, together with the identification of an ADAMTS-4 driven cleavage site between Asn^186^–^187^Gln would again indicate degradation of this peptide as opposed to a reduction in synthesis of fibromodulin. ADAMTS-4 is a pertinent enzyme in the pathogenesis of OA and although fibromodulin has been previously identified as a substrate for ADAMTS-4 [42], this was at the Tyr ^44^–^45^Ala bond [43]. Degraded fragments of the core fibromodulin protein have been observed in OA cartilage [44] and with age [45]. Removal of this portion of fibromodulin would result in weaker interactions of collagen fibers with surrounding structures. This illustrates the potential usefulness of MALDI-MSI in identifying and spatially resolving novel cleavage sites with pathological relevance. Indeed, using two different methodological approaches, this study indicates that cleavage of the fibromodulin peptide ELHLDHNQISR and the biglycan peptide NHLVEIPPNLPSSLVELR is disease, and not age, related. Insights such as this may aid in the understanding of the age-related, but not age-distinct, disease OA.

A number of tentative OA markers were detected. The intensity of the differences in abundance of peptides with *m/z* 1366.5 and 1349.7 in young, old and OA samples found their abundance increased in OA as detected by MALDI-MSI imaging. The *m/z* 1349.7 had been identified from human studies [9] as being derived from fibronectin and its sequence homolog confirmed between the horse and man. Furthermore, we identified the fibronectin peptide *m/z* 1401.7 as being more abundant in OA cartilage compared to young but not old cartilage. In a human OA MALDI-MSI study this peptide was elevated in OA [9]. Here, the fibronectin peptide *m/z* 1401.7 appears to be affected by age-related changes. Fibronectin, an ECM glycoprotein, and fibronectin fragments have been associated with enhanced levels of catabolic cytokines and up regulation of MMPs involved in both normal homeostasis and arthritic diseases. Fibronectin fragments and fibronectin-aggrecan complexes have previously been suggested as biomarkers of OA [46,47]. Therefore, these peptides may provide promising biomarkers of OA as they are not affected by age-related changes. Fibronectin may provide a key species for potential diagnostic and drug targets. The increase in some fibronectin peptides identified in OA cartilage is most likely to be due to an increase in synthesis, according to other authors [9].

The protein melanoma inhibitory activity protein 3 (MIA3) was also identified for the first time in MALDI-MSI studies of cartilage. This is a secreted protein expressed by chondrocytes with a fundamental role in maintaining the chondrocyte phenotype [48]. A similar distribution of a peptide with the same mass as MIA3, *m/z* 1186.74 in young, old and OA tissue was evident (data not shown) which was not surprising as even mature chondrocytes secrete MIA3 [49].

This study undertook analysis on equine cartilage from the metacarpophalangeal joint which is a high motion joint similar to the knee in man. However, cartilage from different joints and different species may represent a different set of matrix changes; hence, it was not surprising that there were some key differences between this study and a previous MALDI-MSI study in end stage OA human cartilage [50]. Although previous studies enabled differential peptide resolution between superficial and deep layers of cartilage to be identified, this was not possible using equine cartilage from the metacarpal joint. This was due to the thickness of the equine cartilage from the metacarpophalangeal III bone which measures 400 to 450 μm, considerably thinner than the human cartilage used. The cartilage from the stifle joint of the horse varies in thickness from 1,760 to 2,215 μm [51] and may provide an alternative source of cartilage for studies attempting to resolve changes in superficial and deep layers of equine cartilage. Secondly, there was no evidence for heterogeneity of peptide distribution that had been previously identified in OA cartilage. This was probably because the former study used severe OA cartilage while here only mild histological changes were evident.

## Conclusions

The *ex vivo* imaging of aged and diseased cartilage provided ‘label-free’ and stain-free information about its biomolecular composition. We identified a number of potential degradation sites in some key ECM proteins as well as markers of cartilage aging. With the resolving power of MALDI-MSI certain to increase in the future and improved methods to identify peptides *in situ*, further MALDI-MSI studies using a greater age range may help in understanding why aging cartilage is more prone to OA.

## Abbreviations

ADAMTS: a distintegrin and metalloproteinase with thrombospondin motifs; COMP: cartilage oligomeric matrix protein; DA: discriminant anlysis; DDA: data dependant analysis; DF: discriminant function; ECM: extracellular matrix; LC: liquid chromatography; MALDI: matrix assisted desorption ionization; MIA: melanoma inhibitory activity protein; MMP: matrix metalloproteinase; MS: mass spectrometry; MS/MS: tandem mass spectrometry; MSI: mass spectrometry imaging; OA: osteoarthritis; PC: principal component; PCA: principal component analysis; TOF-SIMS: time of flight secondary ion mass spectrometry; 95% CI: 95% confidence interval.

## Competing interests

The authors declare they have no competing interests.

## Authors’ contributions

MJP, BCP, PDC and RMAH conceived and designed the study. MJP and BCP undertook the acquisition and analysis of data. GBE contributed to the analysis and interpretation of the data. All authors contributed to the drafting of the article. All authors read and approved the final manuscript.

## Supplementary Material

Additional file 1 **Microscopic (A) and macroscopic (B) evaluation of normal and OA cartilage samples.** Tables containing gross and microscopic scores for the normal old and OA cartilage. Click here for file

Additional file 2 **Combined spectrum of representative digested equine young, old and OA samples reveal different profiles.** Representative spectrum from each group. Click here for file

Additional file 3 **A) PCA scatter plot B.** Discriminant function plot. PCA scatter plot of the first 20 principal components. B) DF1 and DF2 scores. Click here for file

Additional file 4 **Distribution and intensity of the tentative OA marker peptide *m/z *1366.5.** Biomap image and histogram for a *m/z* 1366.5 peptide in representative samples of young, old and OA samples. Click here for file
