# Supplementary material for: Matrix assisted laser desorption ionization mass spectrometry imaging identifies markers of ageing and osteoarthritic cartilage
Source: Arthritis Res Ther. 2014 May 9;16(3):R110. doi: 10.1186/ar4560 (PMC4095688; doi:10.1186/ar4560)
Supplement: Additional file 1 — Microscopic (A) and macroscopic (B) evaluation of normal and OA cartilage samples. Tables containing gross and microscopic scores for the normal old and OA cartilage. [file ar4560-S1.pdf]

Supplementary Table 1. Microscopic (A) and macroscopic (B) evaluation of normal and OA cartilage samples. Numbers in parenthesis relate to the maximum score for that characteristic with higher scores equating to more severe changes.

A

Donor	Structure (/9)	Cellularity (/4)	Cloning (/4)	Total (/17)
Normal 1	0	0	0	0
Normal 2	0	0	0	0
Normal 3	0	0	0	0
OA 1	3	1	1	5
OA 2	2	2	1	5
OA 3	1	3	1	5

B

Donor	Wear lines (/3)	Erosions (/3)	Palmar arthrosis (/3)	Total (/9)
Normal 1	0	0	0	0
Normal 2	0	0	0	0
Normal 3	0	0	0	0
OA 1	0	1	1	1
OA 2	0	1	1	1
OA 3	0	1	1	1
