# Supplementary material for: Matrix assisted laser desorption ionization mass spectrometry imaging identifies markers of ageing and osteoarthritic cartilage
Source: Arthritis Res Ther. 2014 May 9;16(3):R110. doi: 10.1186/ar4560 (PMC4095688; doi:10.1186/ar4560)

Supplementary Figure 1 Combined spectrum of representative digested equine young, old and OA samples reveal different profiles. A representative spectrum from each group is shown. Examples of peptides specific to each condition are seen in the combined spectra and marked in red boxes.

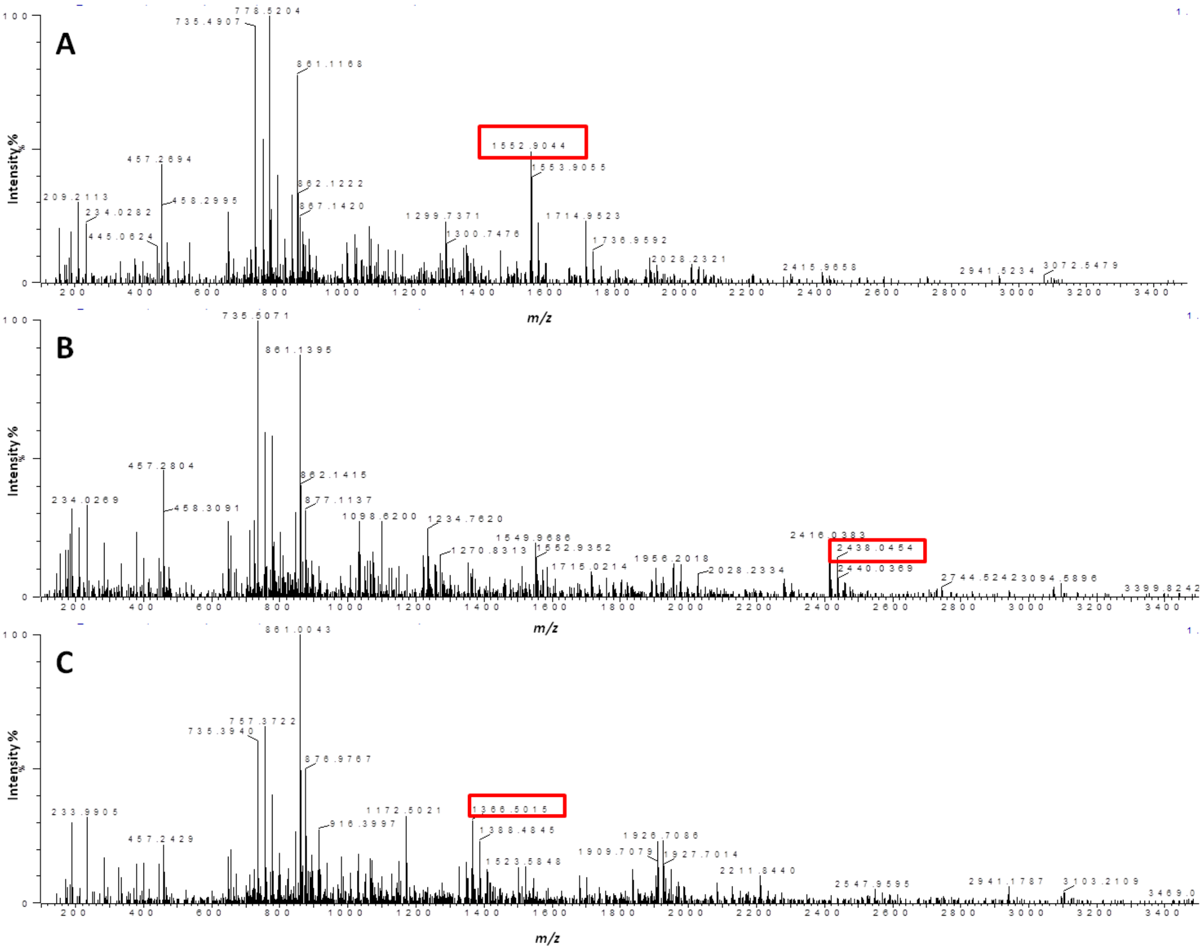

Supplement: Additional file 2 — Combined spectrum of representative digested equine young, old and OA samples reveal different profiles. Representative spectrum from each group. [file ar4560-S2.pdf]
