# Supplementary material for: Matrix assisted laser desorption ionization mass spectrometry imaging identifies markers of ageing and osteoarthritic cartilage
Source: Arthritis Res Ther. 2014 May 9;16(3):R110. doi: 10.1186/ar4560 (PMC4095688; doi:10.1186/ar4560)

Supplementary Figure 2. A. PCA scatter plot revealed separation when the first 20 principal components only were taken into account. Rings denote the different categories; young; 1, old; 2 or OA; 3.B. DF1 and DF2 scores revealed a similar separation.

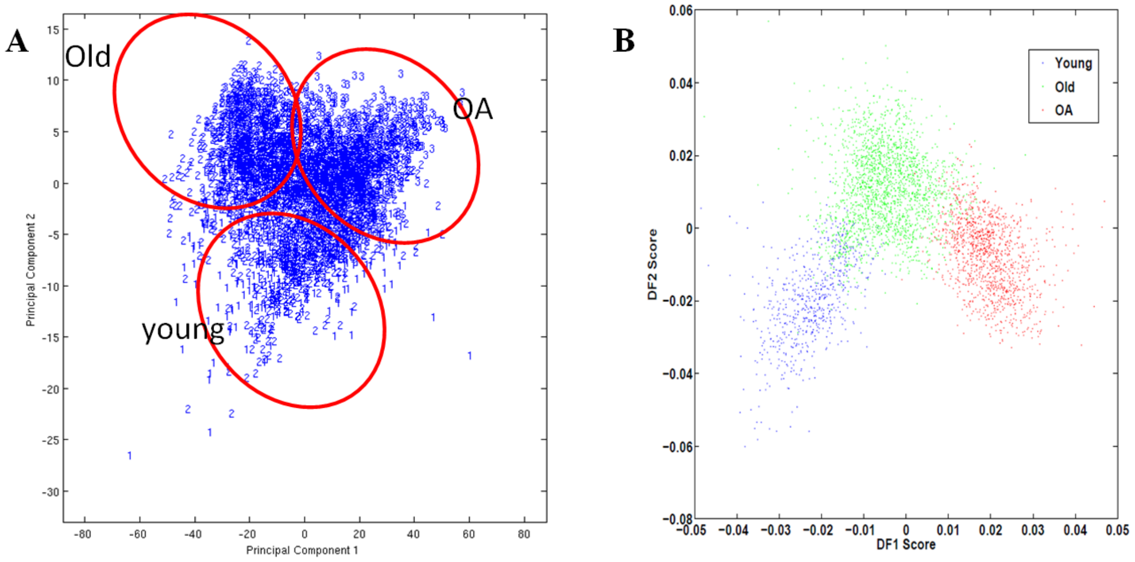

Supplement: Additional file 3 — A) PCA scatter plot B. Discriminant function plot. PCA scatter plot of the first 20 principal components. B) DF1 and DF2 scores. [file ar4560-S3.pdf]
