# Supplementary material for: Matrix assisted laser desorption ionization mass spectrometry imaging identifies markers of ageing and osteoarthritic cartilage
Source: Arthritis Res Ther. 2014 May 9;16(3):R110. doi: 10.1186/ar4560 (PMC4095688; doi:10.1186/ar4560)

Additional File 4. Distribution and intensity of the tentative OA marker peptide *m/z* 1366.5. a) The different intensities and distributions of *m/z* 1366.5 peptides in representative samples of A; young, B; old and C; OA samples after MALDI-MSI experiments (n=3). Scale bar shows normalised intensities. Saturation of the images in panel C; OA samples was evident in order to visualise the intensity in young and old samples. Background was evident in panel B. The intensity values calculated were measured by creating ROI. This prevented the measurement of molecules with the same mass that were outside the tissue. b) Histogram represents the relative mean peak intensities and 95% CI, n=3. * represents P<0.001, young versus OA and old versus OA.

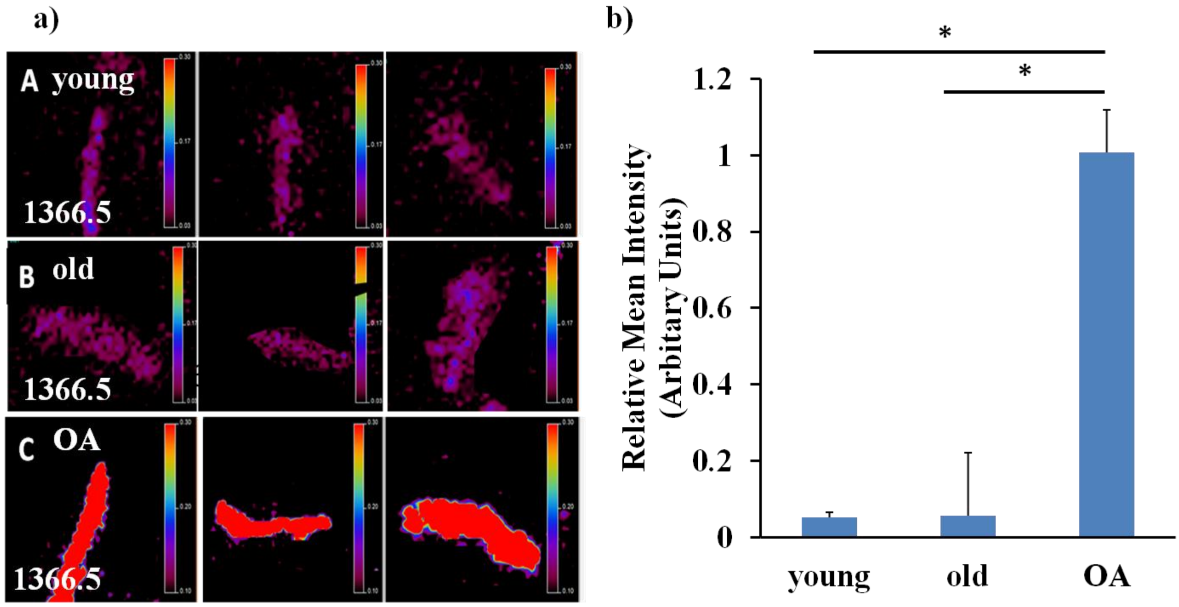

Supplement: Additional file 4 — Distribution and intensity of the tentative OA marker peptide m/z 1366.5. Biomap image and histogram for a m/z 1366.5 peptide in representative samples of young, old and OA samples. [file ar4560-S4.pdf]
